# EGFR blockade prevents glioma escape from BRAF^V600E^ targeted therapy

**DOI:** 10.18632/oncotarget.4014

**Published:** 2015-05-22

**Authors:** Tsun-Wen Yao, Jie Zhang, Michael Prados, William A. Weiss, C. David James, Theodore Nicolaides

**Affiliations:** ^1^ Departments of Pediatrics, University of California San Francisco, San Francisco, CA, USA; ^2^ Departments of Neurological Surgery, University of California San Francisco, San Francisco, CA, USA; ^3^ Departments of Neurology, University of California San Francisco, San Francisco, CA, USA; ^4^ Departments of Neurological Surgery, Feinberg School of Medicine, Northwestern University, Chicago, IL, USA; ^5^ Northwestern Medicine Developmental Therapeutics Institute, Feinberg School of Medicine, Northwestern University, Chicago, IL, USA

**Keywords:** BRAF^V600E^, EGFR, glioma, PLX4720, PTPN9

## Abstract

Mutational activation of BRAF (BRAF^V600E^) occurs in pediatric glioma and drives aberrant MAPK signaling independently of upstream cues. Targeted monotherapy against BRAF^V600E^ displays efficacy in pre-clinical models of glioma, however xenograft tumors adapt rapidly and escape from the growth-inhibitory effects of BRAF-targeted therapy. Here, we show that intrinsic resistance to a BRAF^V600E^ specific inhibitor stems, in part, from feedback activation of EGFR and downstream signaling pathways. BRAF^V600E^ inhibition suppresses MAPK signaling, which in turn downregulates the EGFR phosphatase PTPN9, resulting in sustained EGFR phosphorylation and enhanced EGFR activity. We demonstrated that overexpression of PTPN9 reduces EGFR phosphorylation and cooperates with BRAF^V600E^ inhibitor PLX4720 to reduce MAPK and Akt signaling, resulting in decreased glioma cell viability. Moreover, pharmacologic inhibition of EGFR combined with inhibition of BRAF^V600E^ to reduce growth of glioma cell lines and orthotopic glioma xenograft by decreasing tumor cell proliferation while increasing apoptosis, with resultant significant extension of animal subject survival. Our data support clinical evaluation of BRAF^V600E^ and EGFR targeted therapy in treating BRAF^V600E^ glioma.

## INTRODUCTION

Signaling through mitogen activated protein kinase (MAPK) influences fundamental cellular processes including growth, proliferation, differentiation, migration and apoptosis. MAPK signaling is commonly associated with activation of receptor tyrosine kinases, which in turn and sequentially activate the RAS GTPase, and the RAF kinases (ARAF, BRAF and CRAF). Membrane bound RAF signals through MAP kinase kinases (MEK1 and MEK2) to activate the extracellular signal-regulated kinases (ERK1 and ERK2), which activate downstream transcription factors to drive malignant progression [1]. Among the three RAF isoforms, BRAF is the most potent activator of MEK, and thus has greater oncogenic potential [2]. Aberrant MAPK signaling contributes to at least a third of all cancers [1].

Ninety percent of BRAF mutations substitute glutamic acid for valine at position 600 of BRAF protein, resulting in constitutive kinase activity [3]. Oncogenic BRAF^V600E^ occurs in 50% of metastatic melanomas, 45% of advanced thyroid cancers and in a smaller but substantial number of lung, colorectal, ovarian and brain tumors [3–5]. BRAF^V600E^ mutation is found in 10–67% of pediatric gliomas depending on histopathologic subclassification, and is found less commonly in adult gliomas [6–8] (Supplementary Table 1). Vemurafenib and dabrafenib, BRAF^V600E^ selective inhibitors, are FDA-approved for melanoma therapy [9].

We showed previously that the vemurafenib tool compound, PLX4720, decreased tumor growth and increased survival in animals carrying orthotopic xenografts of BRAF^V600E^-mutant glioma [6]. In addition, Bautista *et al* reported partial and transient response to vemurafenib in two out of three pediatric patients with high grade glioma [10], and Robinson *et al* reported a case of complete regression for recurrent BRAF^V600E^ pediatric giloblastoma multiforme [11]. In combination, preclinical and clinical results have led to an ongoing clinical trial using vemurafenib for treating BRAF^V600E^ glioma (ClinicalTrials.gov Identifier: NCT01748149). Importantly, our preclinical studies using BRAF inhibitor monotherapy revealed that growth inhibition of BRAF^V600E^ glioma was not durable. Here, we investigated the bases for this resistance.

Several lines of evidence indicate that receptor tyrosine kinase (RTK) activation may occur in response to BRAF^V600E^ inhibition [12, 13]. In cancers where RTK signaling is not prominent, such as melanoma, sensitivity to BRAF^V600E^ inhibition can be quite pronounced, suggesting that RTK-based feedback activation may occur more commonly in cancers for which RTK signaling is known to play a prominent role.

Here we test the hypothesis that intrinsic resistance of glioma cells to BRAF^V600E^ inhibitor PLX4720 is due to feedback activation of EGFR and its downstream signaling pathways. In contrast to either monothereapy, combined inhibition of BRAF^V600E^ and EGFR, both *in vivo* and *in vitro*, resulted in efficient silencing of the MAPK pathway, reflected by reduced tumor growth in and extended survival of animal subjects bearing BRAF^V600E^ glioma xenografts. Our data suggests that BRAF^V600E^ and EGFR combination therapy should be considered for clinical evaluation in BRAF^V600E^ glioma.

## MATERIALS AND METHODS

### Cell source and investigational agents

Melanoma cell lines (A375, WM793) were obtained from Dr. Martin McMahon (UCSF); GMB cell lines AM38, DBTRG-05MG and NMC-G1 were obtained from ATCC and the Japanese Tumor Cell Bank Repository. All cell sources were authenticated through DNA fingerprinting using the Promega Powerplex plateform. BT40 pilocytic astrocytoma xenografts were collected under material transfer agreement from Dr Peter Houghton, Nationwide Children's Hospital, USA. DBTRG-05MG cells used for intracranial xenografting were modified by lentiviral infection for stable expression of firefly luciferase to allow *in vivo* bioluminescence imaging [14]. Specific procedures for the preparation of tumor cells from subcutaneous xenografts for transfer to the intracranial compartment, have been previously described [15].

PLX4720 was provided by Plexxikon Inc (Berkeley, CA, USA) and HKI-272 (Niratinib) was purchased from TSZ Scientific LLC (MA, USA). For *in vitro* study, PLX4720 and HKI-272 were dissolved in dimethylsulfoxide (DMSO) at 10 mM. For administration to animal subjects, PLX4720 was dissolved in DMSO/PBS (1:1) at the concentration of 5 g/L, whereas HKI-272 was dissolved in 0.5% hydroxypropyl methylcellulose and 0.2% Tween 80 at a concentration of 8 g/L.

### Cell culture and transfection

All cell lines were maintained in DMEM supplemented with 10% fetal bovine serum, 1% penicillin and streptomycin, and 1% non-essential amino acid. For EGFR knock down experiment, cells were transfected with Dharmacon siGENOME non-target siRNA or EGFR SMARTpool siRNA following manufacturers instruction (Thermo Scientific, MA, USA). For PTPN9 overexpression, cells were transfected with PTPN9 in the pCMV6-AC-GFP plasmid (Origene, MD, USA) using the Amaza Basic Glial Cells Nucleofector Kit (Lonza, Germany) following manufacturers instructions.

### Subcutaneous xenograft

BT-40 pilocytic astrocytoma chunks were subcutaneously implanted into the right flanked of eight week old female non-SCID mice (C.B-17 background, Taconic Farms, Inc). These mice were then randomized to four groups receiving treatment of vehicle control (dimethyl sulfoxide (DMSO) by intraperitonial injection and 0.5% hydroxypropyl methylcellulose plus 0.2% Tween 80 by oral gavage), PLX720 (10 mg/kg) alone, HKI-272 (40 mg/kg) alone, or a combination of PLX4720 and HKI-272. PLX4720 was administered by daily intraperitonial injection whereas HKI-272 treatment was carried out by daily oral gavage. All treatments were initiated when tumor volume reached at 100 mm^3^. The greatest longitudinal diameter (length) and the greatest transverse diameter (width) of tumors were measured by caliper every second day, and tumor volume was calculated using the equation ½ × (length × width^2^) [16]. Mice were monitored daily and euthanized upon significant tumor burden (length > 20 mm), weight loss > 15%, or symptoms related to tumor burden, such as skin ulcer.

### Intracranial tumor implantation in athymic mice

Intracranial tumor cell injection was performed on five-week-old female athymic mice (nu/nu, homozygous; Simonsen Laboratories, Gilroy, CA) following protocols approved by the University of California San Francisco Institutional Animal Care and Use Committee. In brief, mice were anesthetized by intraperitoneal injection of ketamine (100 mg/kg) and xylazine (10 mg/kg) and then were injected with 3 μL of DBTRG-FL cell suspension (3 × 10^5^ cells total) into the right caudate putamen [15].

### Bioluminescence imaging (BLI) of intracranial tumor growth

In preparation for BLI, mice were anesthetized with ketamine (100 mg/kg) and xylazine (10 mg/kg) followed by intraperitoneal inijection of 150 mg/kg of luciferin (D-luciferin potassium salt; Gold Biotechnology, St. Louis, MO). Ten minutes post- luciferin injection, tumor bioluminescence level was measured using an IVIS Lumina imaging station (Caliper Life Sciences, Alameda, CA). Regions of interest, defined by the Living Image software (Caliper Life Sciences, Alameda, CA), were recorded as photons per second per steradian per square centimeter. Mice were imaged twice weekly one week post- intracranial tumor cell injection.

### Immunoblotting analysis

Proteins were extracted from cells and tissues using cell lysis buffer (Cell Signaling) supplemented with proteinase (Roche) and phosSTOP phosphatase inhibitor cocktail (Roche). Proteins were separated by SDS-PAGE and transferred onto polyvinylidene difluoride membranes, which was then probed with primary antibodies followed by horseradish peroxidase-conjugated secondary antibody, and visualized by ECL (Pierce). Antibodies specific for p-MEK 1/2, total MEK 1/2, p-ERK, p-AKT (Ser473), total AKT, PTPN9, and beta-actin were obtained from Cell Signaling Technologies. Total EGFR and total ERK antibodies were purchased from Santa Cruz Biotechnology. Antibody specific for p-EGFR (1173) was obtained from Novus Biologicals, and antibodies specific for Beta-Tubulin was from Milipore.

### Immunohistochemistry

Resected mouse brains were fixed in 10% buffered formalin, then paraffin-embedded and sectioned for hematoxylin and eosin (H&E) staining and immunohisto- chemical (IHC) analysis. To determine Ki-67 reactivity, unstained sections were processed with a Ventana BenchMark XT automated system followed by antigen retrieval that involves a 30 min incubation in Cell Conditioning Solution (CC1) (Ventana Medical Systems) at 92°C. The slides were then stained with anti- Ki-67 (Ventana) antibody for 16 min at 37°C. Ki-67 positive cells were counted in 10 high-powered fields within the tumor, with percent positive cells averaged for all fields associated with a specific drug treatment and subjected to statistical analysis as described below.

### Cell viability assay

Cells were seeded onto 48-well plates at 2500 to 3000 cells per well. After 16 hours of seeding, cells were treated with PLX4720 and/or HKI-272 for 3 days. Cell viability was determined by WST-1 assay (Roche) according to manufacturer's instructions. 450 nm absorbance was measured using a microplate reader (Gen5, BioTek), with background reading at 800 nm subtracted.

### Cell cycle analysis

Cells were treated with or without drugs for 24 hours before cell cycle analysis. The trypsinized cells were washed with PBS and fixed with ice-cold 70% ethanol overnight followed by staining with propidium iodide (20 μg/ml) in PBS containing RNaseA (0.4 mg/ml) (Invitrogen). Fluorescence levels (488nm excitation) were measured by FACSCalibur (Becton Dickinson), and data was analyzed using the ModFit software (Verity).

### Statistical analysis

All statistical analysis was performed using PRISM 5, version 5.03 (GraphPad Software). For survival analysis, a log-rank (Mantel-Cox) test was employed to determine the significance. Animals that died during anesthesia or oral gavages were excluded from survival analyses. A 2-tailed unpaired *t*-test was applied for all other statistical analyses.

## RESULTS

### EGFR is highly expressed and activated in glioma cells

**Figure 1:**
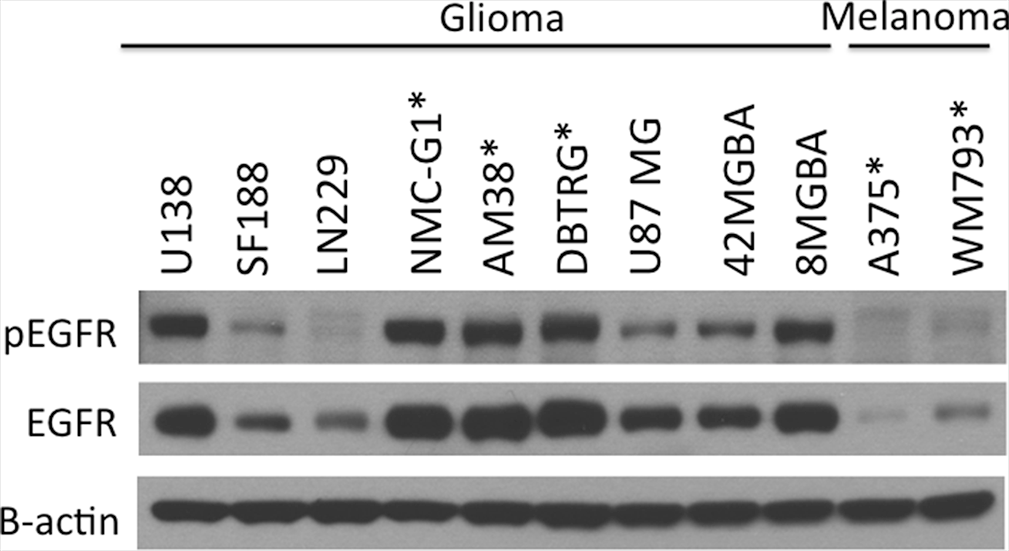
EGFR is highly expressed and activated in glioma cells compared to melanoma cell lines A panel of melanoma and glioma cell lines was examined for EGFR phosphorylation and expression levels by immunoblotting. EGFR was highly activated and readily detectable in most glioma cell lines compared to melanoma cells, which has low levels of EGFR expression. Cell lines expressing BRAF^V600E^ are marked with asterisk.

EGFR expression and signaling is known to be important to the pathobiology of malignant glioma [17]. We compared EGFR expression and activation in human glioma cell lines, and in relation to several melanoma cell lines representative of a cancer in which RTK signaling is not prominent. Both total and phosphorylated EGFR were expressed at consistently higher levels in glioma as compared with melanoma cells (Figure 1 and Supplementary Figure 1). Genetic characteristics of the cell lines tested are described in Supplementary Table 2.

### Combined EGFR and BRAF^V600E^ inhibition results in tumor regression in a subcutaneous xenograft model of BRAF^V600E^ glioma

**Figure 2:**
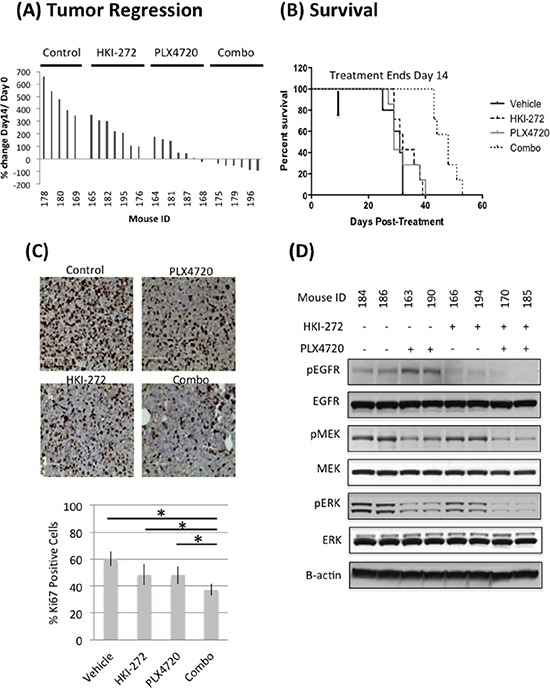
Combined inhibition of EGFR and BRAF^V600E^ significantly reduces tumor growth A. and prolonged animal survival B. in a subcutaneous BT-40 xenograft model Mice carrying subcutaneous BT-40 xenografts were treated with PLX4720 (10 mg/kg, intraperitoneal injection), HKI-272 (40 mg/kg, oral gavage) or a combination of both drugs daily for two weeks. Treatment was initiated when the tumor size reached 100 mm^3^. (A) Percent change in tumor volume after two weeks of daily drug treatment. The data was normalized against tumor size at Day 0 of treatment. Reduction in tumor size was observed in mice treated with a combination of PLX4720 and HKI-272, but not in the vehicle control or single drug treatment groups. (B) Survival profile of the same cohort of mice, which were euthanized due to increasing tumor burden. Mice received a combination of PLX4720 and HKI-272 had significantly extended survival compared to vehicle control (*p* = 8.65E-7) or single drug treatments (*p* value of combo vs PLX4720 = 0.0005; combo vs HKI-272 = 8.74E-6). **C.** Three mice from each group were euthanized 2 hours after the last day of a 3-day daily treatment. Tumors were resected and stained for Ki-67 as an indication of the cell proliferative status. Samples from the combination treatment group were significantly less proliferative compared to vehicle control (*p* = 0.0002) or single drug treatment groups (*p* value of combo vs PLX4720 = 0.0007; combo vs HKI-272 = 0.0159). **D.** The same tumors were also analyzed by immunoblotting to determine the extent of pathway activation. Tumors from the PLX4720 single drug treatment group showed elevated EGFR phosphorylation, whereas samples from mice treated with a combination of PLX4720 and HKI-272 has the least MEK and ERK activation levels. Phospho-Akt was undetectable in these tumors.

HER family proteins play key roles in stimulating ERK signaling upon BRAF^V600E^ inhibition [12, 13]. We therefore asked whether the efficacy of BRAF^V600E^ inhibition could be enhanced by simultaneous EGFR inhibition. We treated mice bearing a BRAF^V600E^ heterozygous mutant pediatric glioma, BT40, with BRAF^V600E^ specific inhibitor PLX4720 (10 mg/kg), EGFR inhibitor HKI-272 (40 mg/kg), or a combination of PLX4720 (10 mg/kg) and HKI-272 (40 mg/kg) for 14 days. Mice receiving PLX4720 and HKI-272 monotherapy showed reduced tumor growth compared to the vehicle control, although no significant benefit was observed in survival (defined by sacrifice of mice required upon tumors reaching maximum tumor burden defined by our animal use protocol) (Figure 2A and 2B). Co-administration of PLX4720 and HKI-272 resulted in a striking regression of tumor volume, with nearly complete regression observed in several mice receiving combination treatment (Figure 2A). As expected, the dramatic reduction in tumor size resulted in significant survival benefit for mice receiving combination treatment (Figure 2B). A wildtype BRAF model was excluded from our analyses as it has been shown that in contrast to BRAF^V600E^ cells, PLX4720 promotes wildtype BRAF glioma growth due to transactivation of wild type BRAF [18].

We next analyzed BT40 xenografts obtained from mice following just three days of treatment for molecular indicators of response to mono- and combination therapy. Immunohistochemical analysis of tumors from mice treated with PLX4720 and HKI-272 showed significantly less positivity for the proliferation marker Ki67 (Figure 2C), compared to vehicle or monotherapy groups. Immunoblotting of protein extracted from frozen portions of the same tumors revealed that PLX4720 monotherapy group had elevated levels of EGFR phosphorylation relative to EGFR phosphorylation in tumors from any other treatment group, suggesting that EGFR is activated by BRAF^V600E^ inhibition (Figure 2D). Since both EGFR and BRAF can drive MAPK signaling, we next analyzed MAPK activation. PLX4720 and HKI-272 co-treated tumors had low levels of MEK and ERK phosphorylation as compared to monotherapy controls (Figure 2D). Molecular indication of anti-tumor activity for either monotherapy was supported by reduced MEK/ERK and/or EGFR phosphorylation in PLX4720 and HKI-272 treated groups. Phospho-Akt was undetectable in any of the tumor samples (data not shown). PLX4720 and HKI-272 concentrations in BT40 subcutaneous xenografts receiving combination treatment were determined as 43.73 ± 17.43 μM and 2.02 ± 0.39 μM, respectively, and both of these well-exceed published *in vitro* kinase assay IC50 values for PLX4720 (13 nM) against BRAF^V600E^, and HKI-272 (92 nM) against EGFR [19, 20]. Interestingly, while the tumor PLX4720 concentration in the monotherapy group (36.32 ± 13.16 μM) resembles those from the animals treated with a combination of PLX4720 and HKI-272 (*p* = 0.34), tumor HKI-272 concentration was significantly higher in the combination treatment group compared to the HKI-272 alone group (1.54 ± 0.03 μM) (*p* = 0.036), suggesting an enhanced uptake of HKI-272 when administered in combination with PLX4720. Although the associated mechanism for increased uptake is yet to be determined, the drug concentration results were consistent with the analysis of EGFR phosphorylation, which showed a more substantial inhibition of phosphorylation when cells were co-treated with HKI-272 and PLX4720 (Figure 2D).

### Co-inhibition of BRAF^V600E^ and EGFR reduces intracranial tumor growth and increases overall animal subject survival

**Figure 3:**
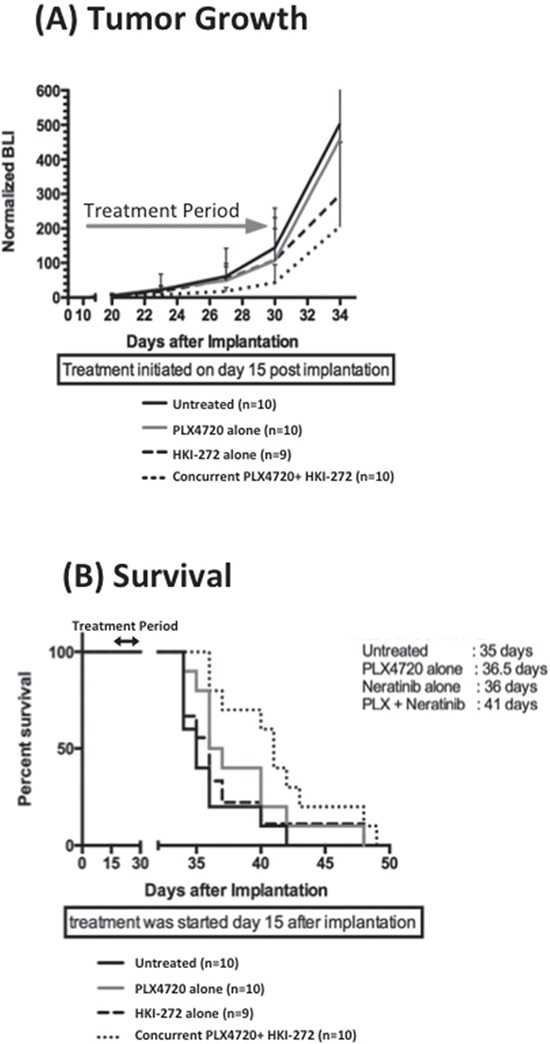
Combined EGFR and BRAF^V600E^ inhibition significantly reduces intracranial BRAF12 DBTRG-FL tumor growth and prolonged animal survival Ten mice were treated with PLX4720 (10 mg/kg, intraperitoneal injection), HKI-272 (40 mg/kg, oral gavage) or a combination of both drugs daily for two weeks started 15 days post intracranial implantation of 3 × 10^5^ BRAF12 DBTRG-FL cells. Quantitative BLI show a substantial anti-proliferative effect **A.** and significantly extended survival **B.** of mice treated with a combination of PLX4720 and HKI-272 compared to untreated control. *P* = 0.055 and 0.0044 for proliferation and survival respectively for the combination treatment vs control group. In contrast, mice received single drug treatment showed no significant survival advantage or anti-proliferative effect compared to the untreated control mice.

To determine whether enhanced efficacy of BRAF^V600E^ + EGFR combination therapy is evident against orthotopic BRAF^V600E^ xenografts, mice were injected intracranially with DBTRG-05MG BRAF^V600E^ cells (heterozygous for BRAF^V600E^), modified with a luciferase reporter, and then treated using the same 14 day regimes as above. DBTRG-05MG was chosen for this orthotopic study because BT40 failed to proliferate as intracranial xenograft. Bioluminescence imaging of intracranial tumors showed the slowest tumor growth rate in mice treated with a combination of PLX4720 and HKI-272 (Figure 3A): a result consistent with combination treatment group mice experiencing the greatest survival benefit (*p* = 0.004) (Figure 3B). In contrast, no significant survival benefit was observed for the PLX4720 (*p* = 0.13) or HKI-272 (*p* = 0.53) monotherapy groups relative to untreated control (Figure 3B).

Analysis of intracranial tumor for drug content revealed less than 50 nM HKI-272 in intracranial DBTRG-FL tumor, more than 40-fold less than for subcutaneous tumor, and also less than the *in vitro* kinase assay IC50 value for this drug against EGFR. The intracranial tumor concentration of PLX4720 was also substantially less than that measured in the subcutaneous tumor (2.25 ± 1.16 μM) but still in excess of the IC50 for PLX4720 against BRAF^V600E^ (13 nM). The plasma HKI-272 (0.95 μM and 1.03 μM for flanked and intracranial tumors respectively) and PLX4720 (131.08 μM and 155.85 μM for flanked and intracranial tumors respectively) were similar between our flanked and intracranial xenograft models, suggesting that the reduced intracranial tumor drug concentration is most likely due to variations in tumor uptake.

### PLX4720 induces feedback activation of EGFR and its downstream signaling

**Figure 4:**
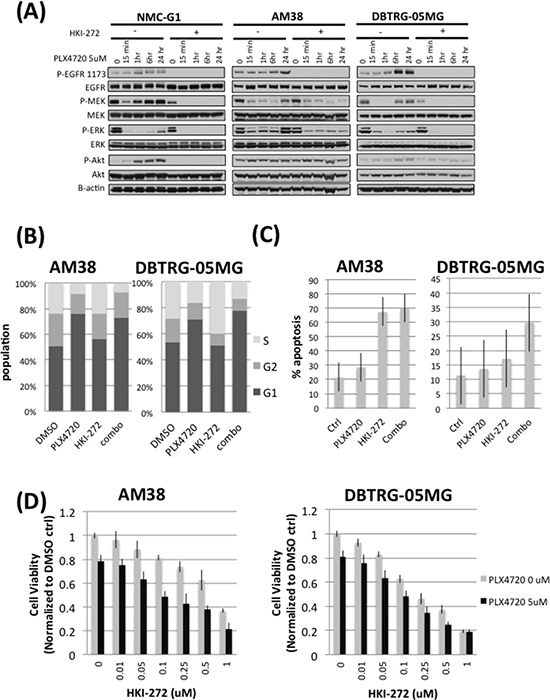
Combined EGFR and BRAF^V600E^ inhibition prevents rebound of MAPK and Akt signaling, elevates G1 phase cell cycle arrest, reduces cell viability and increases apopsotis in BRAF^V600E^ glioma cells **A.** Three BRAF^V600E^ glioma cell lines, NMC-G1, AM38 and DBTRG-05MG were subjected to a 24 hour time course treatment with 5 μM PLX4720 in the presence or absence of 1 μM HKI-272. These cells were serum starved for 16 hours before being stimulated with 10% FBS and harvested for immunoblotting analysis. Cells treated with PLX4720 alone showed an initial suppression of MEK and ERK phosphorylation followed by a profound rebound of MAPK pathway activation as early as one hour post-PLX4720 treatment. Elevated levels of EGFR phosphorylation were also observed in the PLX4720 treated cells. The extent of Akt phosphorylation increased upon PLX4720 treatment, although the extent varies between cell lines. In contrast, no reactivation of EGFR, MEK, ERK or Akt was observed in cells pre-treated with HKI-272. **B.** AM38 and DBTRG-05MG cells were treated with 1 μM HKI-272 and/or 5 μM PLX4720 as indicated for 24 hours before being subjected to cell cycle analysis by propidium iodide staining. Combined EGFR and BRAF^V600E^ inhibition results in elevated G1 phase population (AM38 *p* = 0.001; DBTRG-05MG *p* = 0.0003). **C.** Cells were treated with 1 μM HKI-272 and/or 5 μM PLX4720 as indicated for 72 hours. Apoptosis was determined by annexin V staining. In both AM38 and DBTRG-05MG cells, HKI-272 treatment alone elicited profound apoptosis (*p* value DMSO vs HKI-272 in AM38 = 7.01E-6; in DBTRG-05MG = 0.02) such that no significant additive effect was observed when combined HKI-272 with PLX4720 (*p* value HKI-272 vs combo in AM38 = 0.416; in DBTRG-05MG = 0.07). **D.** Cells were treated 0 (grey bar) or 5 μM (black bar) PLX4720 in the presence of a titration of HKI-272 for 48 hours. Cells viability was measured by WST-1 assay. PLX4720 treatment alone reduced cell viability to approximately 80%. The cell viability loss was further augmented by HKI-272 treatment.

We examined whether EGFR feedback activation by BRAF^V600E^ inhibition was evident *in vitro*, by treating three BRAF^V600E^ glioma cell lines (BRAF^V600E^ homozygous line AM38 and BRAF^V600E^ heterozygous lines DBTRG-05MG and NMC-G1) with 5 μM PLX4720, 1 μM HKI-272 or a combination of both drugs. PLX4720 treatment increased EGFR phosphorylation, and also activated downstream effectors MEK and ERK, in all three BRAF^V600E^ glioma cell lines, as early as 1 hour post-PLX4720 treatment (Figure 4A). A profound increase in Akt phosphorylation was observed in NMC-G1 cells treated with PLX4720. The activation of EGFR signals through RAS and C-Raf to activate MEK and ERK, as shown by elevated levels of RAS-GTP and p-C-Raf following BRAF inhibition (Supplementary Figure 2). Combining HKI-272 with PLX4720 prevented increased phosphorylation of MEK, ERK and Akt in all lines (Figure 4A). Similar inhibitor effects, on signaling mediator phosphorylation, were observed irrespective of cell stimulation by addition of serum (Figure 4A) or by EGF (Supplementary Figure 2).

### EGFR inhibitor HKI-272 cooperates with PLX4720 to induce cell cycle arrest and viability loss

To investigate if suppression of EGFR feedback activation in PLX4720 treated cells affects cell cycle progression and viability, we treated AM38 and DBTRG-05MG cells with 5 μM PLX4720, 1 μM HKI-272, or a combination of both drugs, and measured cell cycle distributions by propidium iodine incorporation, cell viability by WST-1 assay, and apoptosis by annexin V staining. After 24 hours of drug treatment, PLX4720 induced a substantial accumulation of G1 phase cell population compared to DMSO control (DMSO vs PLX4720 treatment: AM38 *p* = 0.001; DBTRG-05MG *p* = 0.0003). Inclusion of HKI-272 with PLX4720 further increased G1 arrest in DBTRG-05MG cells (PLX4720 vs combination, *p* = 0.02) (Figure 4B). G1 phase cell cycle arrest was accompanied by increased apoptosis and decreased cell viability from PLX4720 + HKI-272 co-treatment. Combined treatment with PLX4720 and HKI-272 induced significant apoptosis in both AM38 and DBTRG-05MG cells, in relation to DMSO treatment (DMSO vs combination treatment in AM38, *p* < 0.001; in DBTRG, *p* = 0.046) (Figure 4C). Consistent with the apoptosis results, following 72 hours of drug exposure, PLX4720 treatment caused a significant reduction in cell viability compared to vehicle control. Cell viability was further diminished by the inclusion of HKI-272 with PLX4720 (Figure 4D). The effect of HKI-272 on PLX4720-associated cell viability was masked due to the potency of HKI-272 when used at a concentration > 0.5 μM in DBTRG-05MG cells.

### EGFR knock down prevents PLX4720 induced MAPK activation and cooperates with PLX4720 to induce cell viability loss

**Figure 5:**
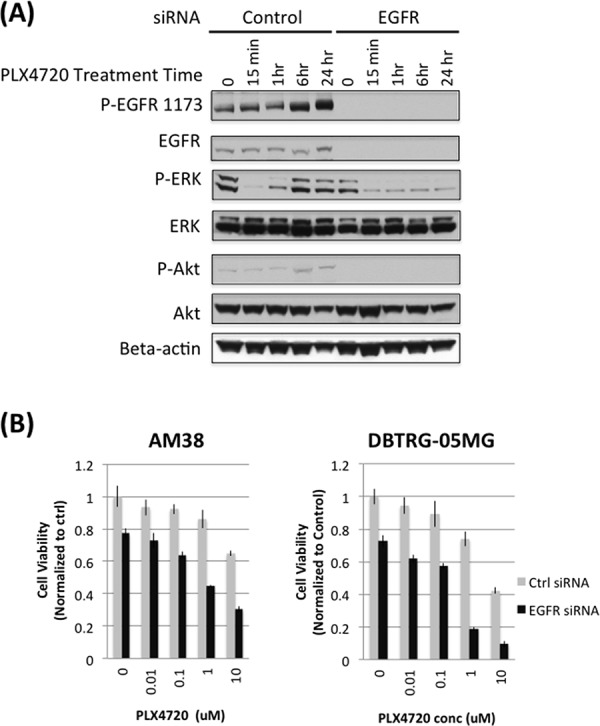
EGFR knock down prevents rebound of MAPK and impairs cell viability in PLX4720 treated glioma cells **A.** AM38 cells were treated with scramble control or EGFR siRNA for 4 hours followed by 5 μM PLX4720 for the period as indicated. Cells were serum starved for 16 hours before being stimulated with 5 nM EGF for 10 min. Rebound of ERK and Akt activation was observed in control, but not in EGFR siRNA treated cells. **B.** Scramble control (grey bar) or EGFR (black bar) siRNA treated AM38 and DBTRG-05MG cells were exposed to 5 μM PLX4720 for 72 hours. Cell viability was measured by WST-1 assay. Cell viability loss induced by PLX4720 was more profound in EGFR knocked down cells compared to control siRNA treated cells.

HKI-272 is an irreversible pan-HER receptor tyrosine kinase inhibitor whose use, in the context of the present study, raises the question of whether HKI-272 effects are due to the inhibition of EGFR only, or is due to combined HER family member inhibition. To address the importance of EGFR inhibition to the effects of HKI-272, we used siRNA knockdown to suppress EGFR expression in AM38 and DBTRG-05MG cells, and then examined siRNA-treated cell response to BRAF^V600E^ pharmacologic inhibition. AM38 and DBTRG-05MG cells treated with scrambled siRNA showed a significant increase in ERK phosphorylation after 6 hours of PLX4720 treatment (Figure 5A). Increased MAPK activation, following treatment with PLX4720, was almost completely blocked by EGFR knock down (Figure 5A). A similar, albeit less pronounced effect was observed for Akt activation.

EGFR siRNA treatment, both with and without PLX4720, decreased cell viability. In the absence of PLX4720, EGFR knock down resulted in 20–30% cell viability loss compared to control siRNA treatment (Figure 5B). Further reduction in cell viability was observed by BRAF^V600E^ inhibition. In the presence of 1 μM PLX4720, EGFR knock down caused 60% and 80% viability loss in AM38 and DBTRG-05MG cells, respectively.

### Hyperactivation of EGFR upon BRAF^V600E^ inhibition coincident with PTPN9 downregulation

**Figure 6:**
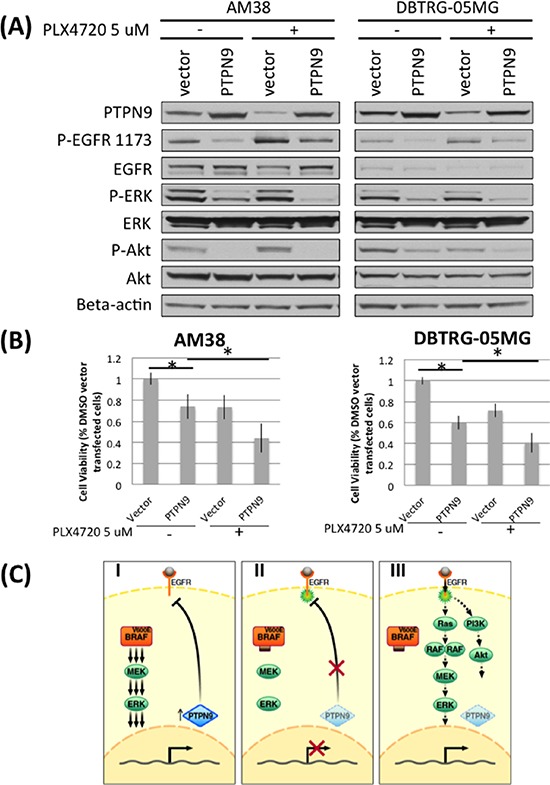
Downregulation of PTPN9 upon PLX4720 treatment correlates with increased EGFR phosphorylation in BRAF^V600E^ glioma cells **A.** AM38 and DBTRG-05MG were transfected with vector or PTPN9 for 24 hours before being treated with 5 μM PLX4720 for 16 hours under serum starved condition. Cells were then stimulated with 5 nM EGF for 15 min before being analyzed for EGFR pathway activation and PTPN9 expression by immunoblotting. Downregulation of the EGFR phosphatase PTPN9 was observed in both cell lines examined when treated with PLX4720, and the level inversely correlates with extent of EGFR phosphorylation. Compared to vector control, PTPN9 overexrpessing cells showed a marked reduction in EGFR phosphorylation and decreased signaling through MAPK and Akt. **B.** Vector or PTPN9 transfected cells were treated with 5 μM PLX4720 for 48 hours. Cell viability was measured by a WST-1 assay. PTPN9 overexpression significantly impaired cell viability in AM38 (*p* = 0.019) and DBTRG-05MG cells (*p* = 0.004). Such viability lost was further augmented by PLX4720 treatment (*p* value vector vs PTPN9 treated with PLX4720 in AM38 = 0.029; in DBTRG-05MG = 0.002). **C.** Schematic representation of intrinsic resistance mechanism to PLX4720 in BRAF^V600E^ glioma. (I) Strong BRAF^V600E^ induced MAPK signaling supports PTPN9 expression, which dephosphorylates and hence inactivates EGFR. (II) MAPK pathway inhibition suppresses PTPN9 expression, resulting in reduced dephosphorylation and therefore activation of EGFR. (III) Overtime, cells reach a new equilibrium where despite lack of BRAF^V600E^ driven signaling, restoration of EGFR activation leads to re-engagement of MAPK and PI3K/Akt pathways.

To define the basis of EGFR feedback activation by PLX4720, we compared the gene expression of known EGFR phosphatases in AM38 and DBTRG cells treated with DMSO or PLX4720 for 12 hours (Supplementary Table 3). Among the genes whose expression was significantly decreased by PLX4720 treatment, the transcript for protein tyrosine phosphatase PTPN9 was of particular interest because of its known EGFR inhibitory activity [21]. Also, PTPN9 is the only candidate phosphatase for which we could readily observe protein down-regulation upon PLX4720 treatment (Figure 6A, Supplementary Figure 3). Additional support for the importance of PTPN9 in the pathobiology of BRAF^V600E^ glioma stems from the observation of a two-fold increase in PTPN9 expression in BRAF^V600E^ pediatric astrocytoma (*n* = 5) relative to BRAF wildtype tumors (*n* = 29) (*p* = 0.001), according to the microarray data described in Schiffman *et al* [7] (Supplementary Figure 4).

To extend the analysis of PLX4720 effects on PTPN9 expression, and to determine if this influences EGFR activity, we compared PTPN9 protein expression and EGFR signaling in PLX4720 treated versus untreated cells. Twenty-four hour treatments with PLX4720 reduced PTPN9 expression in each of three BRAF^V600E^ cell lines, AM38, DBTRG-05MG and NMC-G1 (Figure 6A, Supplementary Figure 3), and increased EGFR phosphorylation. Conversely, overexpression of PTPN9 in these cells markedly reduced EGFR phosphorylation and suppressed MAPK and Akt signaling. PTPN9 overexpression cooperates with PLX4720, causing further silencing of the MAPK and Akt pathways in a similar fashion as observed in EGFR inhibitor HKI-272 or siRNA treated cells.

Furthermore, overexpression of PTPN9 cooperates with PLX4720 to reduce cell viability. PTPN9 expression alone significantly impaired AM38 (*p* = 0.019) and DBTRG (*p* = 0.004) cell viability. The viability loss was further augmented by PLX4720 treatment (*p* value vector vs PTPN9 treated with PLX4720 in AM38 = 0.029; in DBTRG-05MG = 0.002). Collectively, these data support PLX4720 treatment as suppressing PTPN9 expression that, in turn, results in reduced EGFR dephosphorylation and thus enhanced EGFR activity.

## DISCUSSION

BRAF^V600E^ targeted therapy has attracted substantial interest for treating multiple types of cancer, including glioma, due to druggable BRAF^V600E^ kinase activity. However, the extent of tumor response to BRAF^V600E^ inhibitor mono-therapy is likely influenced by cell/tissue of origin and corresponding molecular characteristics. In melanoma, BRAF^V600E^ inhibition results in relatively profound and sustainable ERK inhibition in relation to thyroid or colon cancer [12, 22, 23]. The response of melanoma may be attributable, at least in part, to its limited ability to activate receptor tyrosine kinases when subjected to BRAF^V600E^ inhibition. In thyroid cancer cells, resistance to BRAF^V600E^ inhibitors is largely due to activation of HER3 signaling, as supported by the effect of HER kinase inhibitor lapatinib which prevents MAPK signaling recovery, and sensitizes thyroid cancer cells to RAF inhibitors [12]. In colon cancer cells blockade of EGFR by the antibody cetuximab or by EGFR small molecule inhibitors such as gefitinib or erlotinib proves strongly synergistic with BRAF^V600E^ inhibition in suppressing MAPK signaling and reducing cell viability [13]. Here, we showed that in adult and pediatric glioma cells, inhibition of BRAF^V600E^ results in feedback activation of EGFR that reduces the extent and duration of MAPK pathway inhibition. We further demonstrated that genetic and pharmacological inhibition of EGFR cooperates with PLX4720 in efficient blockade of the MAPK and Akt pathways, resulting in induction of G1 phase cell cycle arrest and loss of cell viability. The combined effect of EGFR and BRAF^V600E^ inhibition is also reflected in our *in vivo* BRAF^V600E^ xenograft models. Mice treated with a combination of BRAF^V600E^ and EGFR inhibitors showed dramatic reduction in tumor growth and extended survival compared to vehicle or single drug treated counterparts. More profound survival benefit would be expected with extended courses of drug treatment, which we are actively pursuing. Collectively, these data support BRAF^V600E^ and EGFR combination therapy as an approach for treating BRAF^V600E^ glioma.

We have shown that downregulation of the EGFR phosphatase PTPN9 upon BRAF^V600E^ inhibition is associated with increased EGFR activation [21]. Conversely, we showed that overexpression of PTPN9 reduces EGFR phosphorylation and cooperates with PLX4720 to reduce MAPK and Akt signaling, resulting in cell viability loss. These data argue that BRAF^V600E^ inhibition suppresses MAPK signaling, which in turn downregulates PTPN9, resulting in sustained EGFR phosphorylation and enhanced EGFR activity (Figure 6C). Further supporting this hypothesis is the two-fold upregulation of PTPN9 expression in BRAF^V600E^ pediatric astrocytoma compared to BRAF wildtype counterparts (*p* = 0.001) [7]. While our data do not exclude the involvement of other phosphatases in this MAPK/EGFR feedback mechanism, our observations are distinct from those observed in epithelial cancers. In melanoma cells, ERK rebound upon BRAF^V600E^ inhibition may be due to reduction of the ERK phosphatase DUSP6 and receptor tyrosine kinase blocker Spry2, which sequesters the Grb2:SOS complex, preventing the binding of receptor tyrosine kinase [23]. In colon cancer cells, initial reduction in ERK signaling by BRAF^V600E^ inhibitor treatment decreases the phosphatase activity of CDC25C, which in turn results in elevated EGFR phosphorylation [22]. In thyroid cancer cells, rebound of ERK signaling upon BRAF^V600E^ treatment may be caused by downregualtion of ERK phosphatase DUSP5 and increased HER3 expression [12]. PTPN9 overexpression *per se* reduces glioma cell proliferation in the absence of BRAF inhibition in our experiments (Figure 6), indicating that PTPN9 may have a global tumor suppressive role. PTPN9 is known to dephosphorylate HER2, STAT3, and VEGFR2, in addition to EGFR, and it's expression is normally tightly regulated [24–26]. Interestingly, inactivating mutations or recurrent chromosomal deletions incorporating PTPN9 in GBM are not found in the TCGA database via cBioPortal, which suggests that PTPN9 might not be a common tumor suppressor gene in glioma [27].

In 2014, the FDA has approved the use of the MEK inhibitor trametinib and BRAF^V600E^ inhibitor debrafenib as combination therapy in BRAF^V600E^ and BRAF^V600K^ melanoma. Such an approach is logical for melanoma, in which rebound of MAPK signaling upon BRAF^V600E^ inhibition is largely mediated through the MAPK pathway. In glioma cells multiple signaling pathways, including those involving MAPK and PI3K/Akt, are simultaneously activated by BRAF^V600E^ inhibitor induced feedback activation of EGFR. Therefore, inhibition of EGFR, and the multiple mitogenic pathways it stimulates, may prove to be more efficacious than targeting a single downstream signaling effector.

EGFR targeted therapy has been disappointing in glioma despite great effort in developing small molecule tyrosine kinase inhibitors (erlotinib, gefitinib, lapatanib, PKI166, neratinib, canertinib, pelitinib), monoclonal antibodies (cetuximab, panitumumab, nimotuzumab), and RNA-based agents (siRNA, antisense oligonucleotides OGX-011) [28]. Similarly, some BRAF^V600E^ monotherapy attempts in glioma have been discouraging [29]. It is therefore interesting and significant that combining EGFR and BRAF^V600E^ therapies may prove an effective anti-glioma strategy. If this therapeutic approach were to be used in treating glioma patients, a factor that may prove critically important in determining its success is therapeutic access to brain and brain tumor. Here, our results showed that heightened concentration of HKI-272 in subcutaneous tumor, relative to intracranial tumor, resulted in almost complete subcutaneous tumor regression when combined with PLX4720. A much lesser concentration of intracranial HKI-272 likely explains the more subtle effects on tumor growth and animal survival in our orthotopic model. The HKI-272/PLX4720 combination therapy outperformed single drug treatments in our intracranial model despite lower brain HKI-272 concentration than the published IC50 values of HKI-272 against EGFR, and also the GI50 values (0.1–1 μM) determined from our *in vitro* cell viability assays (Figure 4D). This suggests that partial inhibition of EGFR is sufficient to contribute to suppression of tumor growth when BRAF^V600E^ is being inhibited. Most available anti-EGFR agents, including HKI-272 used in this study, have poor blood brain barrier penetrance. In the absence of EGFR small molecule inhibitors with excellent brain penetration, it may be necessary to resort to alternative routes of administration or inhibitor packaging approaches for achieving sufficiently high tumor concentrations of inhibitor for patients to benefit from this combination therapy.

Our study suggests that EGFR and BRAF^V600E^ combination therapy might be worth investigating in clinical trial on BRAF^V600E^ glioma, which thus far has poor clinical prognosis under standard radiation and chemotherapeutic regimens [30]. The powerful feedback activation of EGFR induced upon BRAF^V600E^ inhibition explains the incomplete clinical response to BRAF^V600E^ monotherapy in early case series. We have shown that preventing such feedback mechanism by inhibiting EGFR can effectively reduce tumor growth and prolong animal survival. Our data also implicates that the expression and activation levels of EGFR may be a useful clinical biomarker to predict the response to BRAF^V600E^ targeted therapy. Last but not least, as acquired resistance is a common problem of small molecule based anti-cancer therapy, we are dedicated to investigating solutions to prevent or overcome acquired resistance associated with EGFR and BRAF^V600E^ targeted therapy.

## SUPPLEMENTARY FIGURES AND TABLES


