# Supplementary material for: EGFR blockade prevents glioma escape from BRAFV600E targeted therapy
Source: Oncotarget. 2015 May 22;6(26):21993–2005. doi: 10.18632/oncotarget.4014 (PMC4673141; doi:10.18632/oncotarget.4014)
Supplement: Supplementary file 1 [file oncotarget-06-21993-s001.pdf]

SUPPLEMENTARY FIGURES AND TABLES

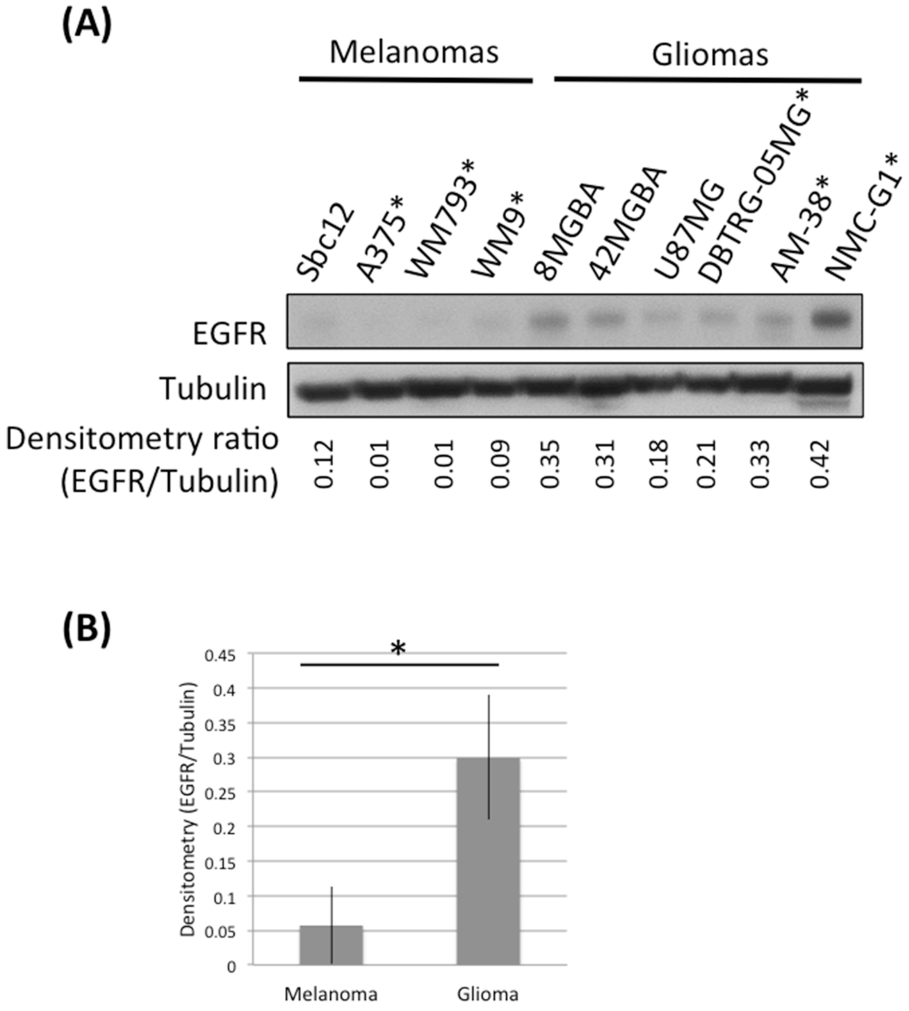

Supplementary Figure 1: EGFR is expressed at a significantly higher level in glioma cells compared to melanoma cell lines. A. A panel of melanoma and glioma cell lines was examined for EGFR expression levels by immunoblotting. EGFR was readily detectable in most glioma cell lines compared to melanoma cells, which have low levels of EGFR expression. Cell lines expressing $BRAF^{V600E}$ are marked with asterisk. **B.** Densitometry analysis of the EGFR immunoblot relative to tubulin loading control (p = 0.013).

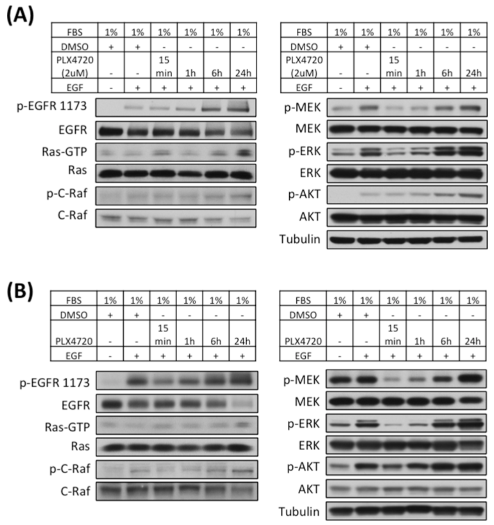

Supplementary Figure 2: PLX4720 induces feedback activation of EGFR and its downstream signaling. A. AM38 cells were treated with 2 µM PLX4720 over the period of time as indicated. Before harvesting, cells were cultured in 1% FBS overnight before being stimulated with 5 nM EGF for 10 min. PLX4720 treatment results in increased phosphorylation of EGFR (Tyr 1173 and Tyr 1068) and its downstream signaling molecules Ras/c-Raf/MEK/ERK and Akt. Similar phenomenon was also observed in DBTRG-05MG cells **B.**

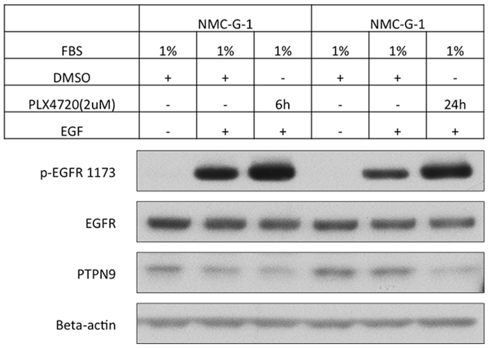

Supplementary Figure 3: Downregulation of PTPN9 upon PLX4720 treatment correlates with increased EGFR phosphorylation in NMC-G1 cell. NMC-G1 cells were treated with 2 µM PLX4720 over a time course of 24 hours. PTPN9 expression and EGFR activation levels were measured by immunoblotting. Cells treated with PLX4720 showed a marked reduction in PTPN9 expression and an increased EGFR phosphorylation during the time period of analysis.

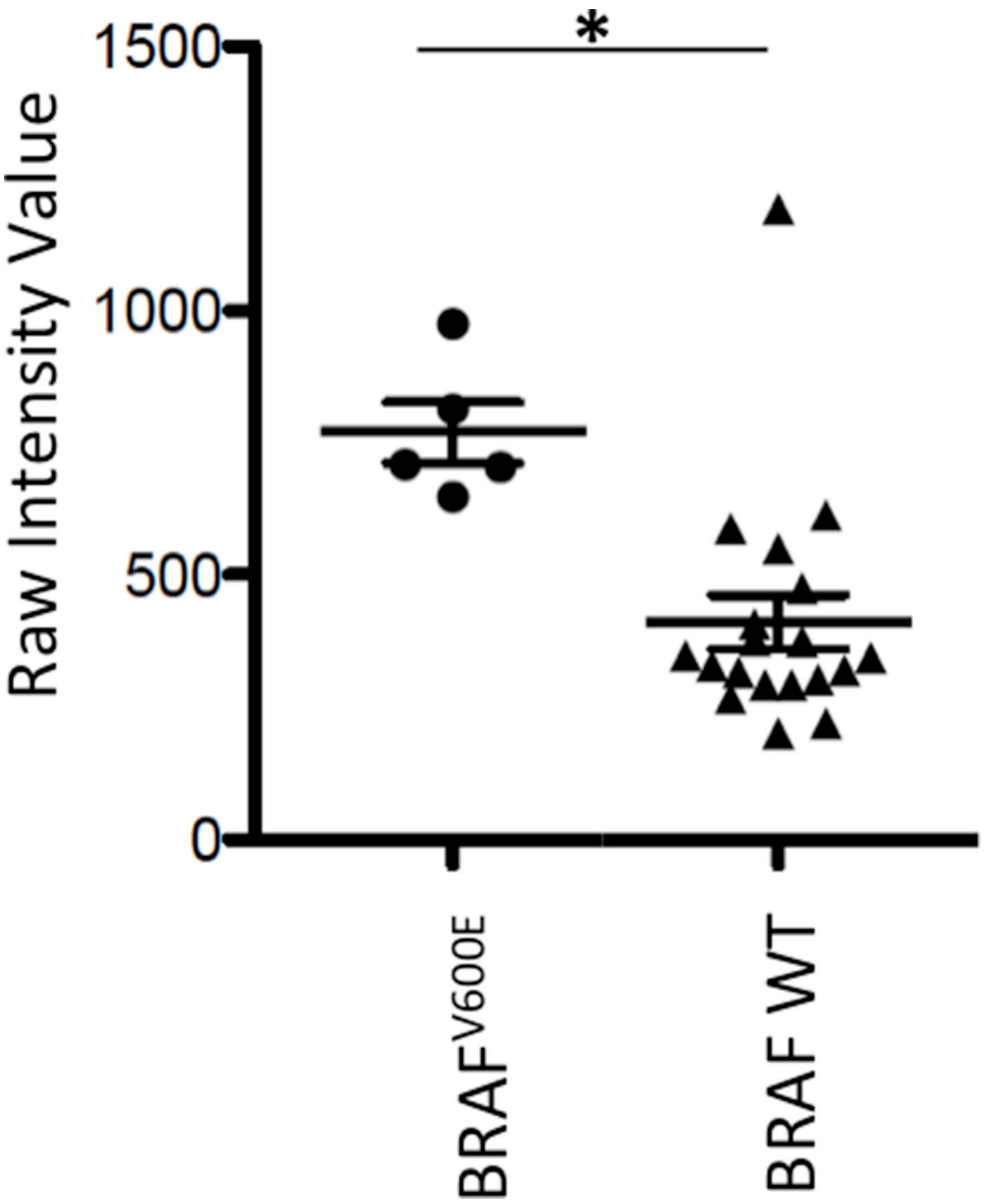

Supplementary Figure 4: Upregulation of PTPN9 in BRAF^{V600E} pediatric astrocytoma. Two-fold increase in PTPN9 expression in BRAF^{V600E} pediatric astrocytoma ($n = 5$) compared to BRAF wildtype counterparts ($n = 29$) ($p = 0.001$). The microarray data was extracted from Schiffman *et al* [7].

Supplementary Table 1: Frequency of BRAF^{V600E} mutations in various glioma subtypes

Frequency of BRAF^{V600E} in Pediatric and Adult Gliomas

**Pediatric (< 18 years old)**	**Grade I- Pilocytic astrocytoma**	
	3/53 (6%)	Pfister et al. J Clin Invest. (2008) 118:1739–1749
	7/75 (9%)	Schindler et al. Acta Neuropathol (2011) 121:397–405
	**Grade I - Ganglioglioma**	
	6/12 (50%)	Dougherty et al. Neuro-Oncology (2010) 12(7):621–630

(*Continued*)

Frequency of $BRAF^{V600E}$ in Pediatric and Adult Gliomas		
	13/28 (46%)	Chappé et al. Brain Pathology (2013) doi: 10.1111/bpa.12048.
	**Grade II - Diffuse astrocytomas**	
	1/13 (8%)	Pfister et al. J Clin Invest. (2008) 118:1739–1749
	0/26 (0%)	Jacob et al. Br J Cancer. (2009) 101(4):722–733.
	**Grade II - Oligodendroglioma**	
	0/2 (0%)	Schindler et al. Acta Neuropathol (2011) 121:397–405
	**Grade II - Pleomorphic xanthoastrocytoma**	
	4/7 (57%)	Dias-Santagata et al. PLoS ONE (2011) 6(3): e17948
	18/26 (69%)	Schindler et al. Acta Neuropathol (2011) 121:397–405
	**Grade III - Anaplastic Astrocytoma**	
	3/9 (33%)	Schiffman et al. Cancer Res (2010) 70:512–519
	2/6 (33%)	Schindler et al. Acta Neuropathol (2011) 121:397–405
	**Grade IV - GBM**	
	2/36 (6%)	Schindler et al. Acta Neuropathol (2011) 121:397–405
	6/44 (14%)	Nicolaides et al. Clin Cancer Res (2011) 17:7595–7604
**Adult (≥ 18 years old)**	**Grade I - Pilocytic astrocytoma**	
	2/22 (9%)	Schindler et al. Acta Neuropathol (2011) 121:397–405
	1/2 (50%)	Ida et al. J Neuropathol Exp Neurol (2012) 71(7):631–639
	**Grade I - Ganglioglioma**	
	11/53 (21%)	Schindler et al. Acta Neuropathol (2011) 121:397–405
	**Grade II - Diffuse astrocytomas**	
	0/53 (0%)	Schindler et al. Acta Neuropathol (2011) 121:397–405
	0/3 (0%)	Ida et al. J Neuropathol Exp Neurol (2012) 71(7):631–639
	**Grade II - Oligodendroglioma**	
	1/62 (2%)	Schindler et al. Acta Neuropathol (2011) 121:397–405
	**Grade II - Pleomorphic xanthoastrocytoma**	
	8/13 (62%)	Dias-Santagata et al. PLoS ONE (2011) 6(3): e17948
	24/38 (63%)	Schindler et al. Acta Neuropathol (2011) 121:397–405
	**Grade III - Anaplastic Astrocytoma**	
	0/52 (0%)	Schindler et al. Acta Neuropathol (2011) 121:397–405
	**Grade IV - GBM**	
	2/34 (6%)	Basto et al. Acta Neuropathol. (2005) 109(2):207–10
	0/79 (0%)	Schindler et al. Acta Neuropathol (2011) 121:397–405

Supplementary Table 2: Genetic lesions of the major known GBM oncogenes and tumor suppressor genes. Data extracted from Michaud *et al* [31] and Ishii *et al* [32].

Cell Lines	Genetic Lesions	Age	Cell type
U138MG	CDKN2A/B homozygous deletion; TP53 homozygous missense; PTEN homozygous splice site	47 yo	Glioblastoma/Grade IV
SF188	Wild type PTEN, p16, p14ARF	8 yo	Glioblastoma Multiforme/Grade IV
LN229	CDKN2A/B homozygous deletion; CDKN2C homozygous deletion; TP53 heterozygous missense	60 yo	Glioblastoma/epithelial cells/Low Grade
NMC-G1	CDKN2A/B homozygous deletion; $BRAF^{V600E}$ heterozygous mutation; NF2 homozygous frameshift	NA	Glioblastoma Multiforme/Grade IV
AM38	CDKN2A/B homozygous deletion; $BRAF^{V600E}$ homozygous mutation	36 yo	Glioblastoma Multiforme/Grade IV
DBTRG-05MG	CDKN2A/B homozygous deletion; $BRAF^{V600E}$ heterozygous mutation; PTEN homozygous deletion	59 yo	Glioblastoma Multiforme/glial cells/ Grade IV
U87MG	CDKN2A/B homozygous deletion; CDKN2C homozygous deletion; PTEN homozygous splice site	44 yo	Glioblastoma/Astrocytoma/epithelial/Grade IV
42MGBA	CDKN2A/B homozygous deletion	63 yo	Glioblastoma Multiforme/epithelial-like
8MGBA	RB1 homozygous deletion; TP53 homozygous missense	52 yo	Glioblastoma Multiforme/epithelial-like

Supplementary Table 3: Protein tyrosine phosphatases with transcript level change in AM-38 cells (PLX4720/Control)

	Log2(fold change)	FoldChange	*P*-value	False Discovery Rate	References
CDC25A	−0.9004	0.5360	0.0001	0.0129	Wang Z et al. J Biol Chem 2002;277(22):19470–5.
PTPN9	−0.7138	0.6100	0.0057	0.1360	Yuan T et al. J Biol Chem 2010;285(20):14861–70.Du WW et al. J Cell Sci 2013;126(Pt 6):1440–53.
PTPN12	−0.6373	0.6430	0.0002	0.0166	Sun T et al. Cell 2011, 144(5): 703–718.
PTPRK	−0.3281	0.7970	0.0115	0.1950	Xu Y et al. J Biol Chem 2005, 280(52): 42694–42700.
